# Supplementary material for: Robust Metabolite Quantification from J-Compensated 2D 1H-13C-HSQC Experiments
Source: Metabolites. 2020 Nov 7;10(11):449. doi: 10.3390/metabo10110449 (PMC7695005; doi:10.3390/metabo10110449)
Supplement: Supplementary file 1 [file metabolites-10-00449-s001.pdf]

Supplemental Material: Robust Metabolite Quantification from J-Compensated 2D ^{1}H-^{13}C-HSQC Experiments

Alexander Weitzel[1,†], Claudia Samol[1], Peter J. Oefner[1], and Wolfram Gronwald[1,*]

[1]Institute of Functional Genomics, University of Regensburg
[*]Correspondence: wolfram.gronwald@klinik.uni-regensburg.de
[†]Current address: Institute of Experimental and Applied Physics, Regensburg

November 6, 2020

S1 Experimental Details

S1.1 Spectrometer Software

We used *Bruker TopSpin 3.1 PL7* for the control of the spectrometer. The pulse sequence *hsqcetgppr*, which is included in *Bruker TopSpin 3.1 PL7*, was used in an altered version by addition of continuous wave presaturation to determine calibration factors and was later used to examine the samples by means of standard HSQC. For the Q-HSQC, we extended this pulse program such that 75 % of scans are acquired with an INEPT delay of $\Delta_1 = 2.94$ ms and 25 % of the scans with an INEPT delay of $\Delta_2 = 5.92$ ms. Furthermore, we replaced the composite ^{13}C inversion pulses by adiabatic inversion pulses of the type *Crp60,0.5,20.1*. This allows the uniform excitation of a larger spectral window of the ^{13}C frequencies. For the QUIPU-HSQC we adapted the pulse sequence established by Mauve *et al.* [1] and replaced the looped solvent inversion by continuous wave presaturation, for which we optimized the offset prior to experiments. Additionally, just as for Q-HSQC, we replaced the composite ^{13}C inversion pulses by adiabatic inversion pulses of the type *Crp60,0.5,20.1*. For each experiment 8 scans were collected in 128 increments.

S1.2 Sample Details

Table S1: Concentration levels of the spiked-in compounds per sample.

**sample**	**acetic acid**	**alanine**	**betaine**	**citric acid**	**creatinine**	**ethanolamine**	**glycine**	**histidine**	**taurine**	**TMAO**
1	0.010	0.020	0.039	0.078	0.156	0.312	0.624	1.248	2.496	4.992
2	0.020	0.039	0.078	0.156	0.312	0.624	1.248	2.496	4.992	0.010
3	0.039	0.078	0.156	0.312	0.624	1.248	2.496	4.992	0.010	0.020
4	0.078	0.156	0.312	0.624	1.248	2.496	4.992	0.010	0.020	0.039
5	0.156	0.312	0.624	1.248	2.496	4.992	0.010	0.020	0.039	0.078
6	0.312	0.624	1.248	2.496	4.992	0.010	0.020	0.039	0.078	0.156
7	0.624	1.248	2.496	4.992	0.010	0.020	0.039	0.078	0.156	0.312
8	1.248	2.496	4.992	0.010	0.020	0.039	0.078	0.156	0.312	0.624
9	2.496	4.992	0.010	0.020	0.039	0.078	0.156	0.312	0.624	1.248
10	4.992	0.010	0.020	0.039	0.078	0.156	0.312	0.624	1.248	2.496

Table S2: Signal properties of the ten test metabolites.

Metabolite	Signal	^{13}C chemical shift [ppm]	^{1}H chemical shift [ppm]	J_{CH} [Hz]	calibration factor
Acetic acid	C2 H2A/H2B/H2C	25.91	1.90	126.5	12.60
Alanine	C2 H2	53.31	3.76	140.7	-
	C3 H3A/H3B/H3C	18.81	1.46	119.8	27.66
Betaine	C2 H2A/H2B	68.76	3.88	140.5	25.41
	C4/C5/C6 methylated	55.92	3.23	147.5	26.98
Citric acid	C2/C4 H2A/H4A	48.26	2.53	126.0	30.12
	C2/C4 H2B/H4B	48.29	2.64	127.1	30.12
Creatinine	C2 H2A H2B	59.07	4.03	141.3	26.98
	C6 H6A/H6B/H6C	32.57	3.05	133.7	22.97
Ethanolamine	C1 H1A/H1B	60.42	3.80	144.4	25.13
	C2 H2A/H2B	44.10	3.12	140.1	23.47
Glycine	C2 H2A/H2B	44.24	3.54	144.3	24.15
Histidine	C2 H2	57.48	3.96	140.8	-
	C3 H3A	30.51	3.22	128.3	-
	C3 H3B	30.62	3.17	117.4	-
	C5 H5	119.58	7.06	193.4	23.55
	C7 H7	138.75	7.83	211.4	-
Taurine	C1 H1A/B	38.18	3.40	142.1	27.55
	C2 H2A/B	50.09	3.23	133.9	25.47
TMAO	C1/C2/C3 methylated	62.15	3.25	141.6	27.48

S2 Supplemental Results

S2.1 Relative errors and relative standard deviations

Table S3: Relative errors (RE) and relative standard deviations (RSD) obtained by the standard HSQC. The true value was assumed to be the concentration value obtained by means of 1D ^{1}H NMR. The RE was then calculated as the difference between the concentration value obtained by standard HSQC and the true concentration value divided by the true concentration value. The concentration estimates were each averaged over the number of experiments (3), if possible. In case the compound was detected in only one or two of the measurements of a triplicate, the corresponding mean value of the two incidents or the single value was used.

conc. level	alanine		creatinine		glycine		taurine		betaine		acetic acid		ethanol-amine		TMAO		histidine		citric acid	
	RE	RSD	RE	RSD	RE	RSD	RE	RSD	RE	RSD	RE	RSD	RE	RSD	RE	RSD	RE	RSD	RE	RSD
1	-	-	-	-	-	-	-	-	-	-	-	-	-	-	-	-	-	-	-	-
2	-	-	-	-	-	-	-	-	-	-	-	-	-	-	-	-	-	-	-	-
3	-	-	-	-	-	-	-	-	0.06	0.16	-	-	-	-	0.28	0.17	-	-	-	-
4	0.19	0.07	0.08	-	0.03	-	0.16	-	0.19	0.06	0.70	-	0.05	-	0.27	0.07	-	-	0.23	-
5	0.19	0.15	0.03	0.07	0.01	0.36	0.03	0.09	0.06	0.07	0.15	0.10	0.06	-	0.26	0.05	0.40	-	0.18	0.27
6	0.11	0.02	0.01	0.05	0.04	0.10	0.06	0.12	0.02	0.02	0.23	0.08	0.02	0.09	0.14	0.01	0.02	0.08	0.02	0.12
7	0.10	0.02	0.05	0.03	0.08	0.05	0.06	0.03	0.04	0.01	0.08	0.07	0.06	0.07	0.15	0.02	0.04	0.03	0.02	0.07
8	0.06	0.01	0.01	0.03	0.06	0.03	0.02	0.01	3E-4	0.02	0.08	0.03	0.06	0.01	0.004	0.01	0.06	0.03	0.04	0.03
9	0.03	0.01	0.003	0.03	0.02	0.03	0.02	0.01	0.05	0.01	0.13	0.02	0.09	0.01	0.02	0.002	0.01	0.01	0.06	0.01
10	0.04	0.01	0.02	0.002	0.003	0.01	0.01	0.01	0.04	3E-4	0.11	0.01	0.09	0.01	1E-4	0.003	0.02	0.02	0.06	0.01

Table S4: Relative errors (RE) and relative standard deviations (RSD) obtained by the Q-HSQC. The true value was assumed to be the concentration value obtained by means of 1D ^{1}H NMR. The RE was then calculated as the difference between the concentration value obtained by Q-HSQC and the true concentration value divided by the true concentration value. The concentration estimates were each averaged over the number of experiments (3), if possible. In case the compound was detected in only one or two of the measurements of a triplicate, the corresponding mean value of the two incidents or the single value was used.

conc. level	alanine		creatinine		glycine		taurine		betaine		acetic acid		ethanol-amine		TMAO		histidine		citric acid	
	RE	RSD	RE	RSD	RE	RSD	RE	RSD	RE	RSD	RE	RSD	RE	RSD	RE	RSD	RE	RSD	RE	RSD
1	-	-	-	-	-	-	-	-	-	-	-	-	-	-	-	-	-	-	-	-
2	-	-	-	-	-	-	-	-	0.16	-	-	-	-	-	-	-	-	-	-	-
3	-	-	0.45	-	-	-	-	-	0.003	-	-	-	-	-	0.07	0.24	-	-	-	-
4	0.05	0.19	0.08	0.08	0.55	-	-	-	0.18	0.10	0.22	0.04	0.07	-	0.59	0.11	-	-	1.23	-
5	0.17	0.01	0.03	0.11	0.01	0.03	0.04	0.12	0.06	0.02	0.25	0.18	0.15	0.07	0.44	0.07	0.51	-	0.41	-
6	0.003	0.14	0.07	0.06	0.12	0.01	0.02	0.06	0.04	0.03	0.27	0.03	0.09	0.07	0.32	0.04	0.04	0.10	0.002	0.01
7	0.02	0.03	0.06	0.04	0.05	0.03	0.07	0.002	0.10	0.01	0.26	0.09	0.16	0.02	0.28	0.02	0.15	0.06	0.01	0.11
8	0.04	0.01	0.13	0.01	0.01	0.01	0.10	0.01	0.08	0.02	0.25	0.03	0.15	0.05	0.13	0.002	0.22	0.01	0.002	0.01
9	0.05	0.02	0.09	0.01	0.05	0.01	0.10	0.01	0.14	0.003	0.24	0.02	0.22	0.01	0.12	0.002	0.22	0.02	0.01	0.01
10	0.06	0.01	0.08	0.01	0.06	0.002	0.11	0.003	0.12	0.01	0.24	0.01	0.18	0.01	0.13	1E-4	0.14	0.01	0.01	0.01

Table S5: Relative errors (RE) and relative standard deviations (RSD) obtained by the QUIPU-HSQC. The true value was assumed to be the concentration value obtained by means of 1D ^{1}H NMR. The RE was then calculated as the difference between the concentration value obtained by QUIPU-HSQC and the true concentration value divided by the true concentration value. The concentration estimates were each averaged over the number of experiments (3), if possible. In case the compound was detected in only one or two of the measurements of a triplicate, the corresponding mean value of the two incidents or the single value was used.

conc. level	alanine		creatinine		glycine		taurine		betaine		acetic acid		ethanol-amine		TMAO		histidine		citric acid	
	RE	RSD	RE	RSD	RE	RSD	RE	RSD	RE	RSD	RE	RSD	RE	RSD	RE	RSD	RE	RSD	RE	RSD
1	-	-	29.19	-	-	-	46.80	-	32.46	-	-	-	54.12	0.34	-	-	-	-	-	-
2	-	-	10.56	-	13.95	-	15.94	-	39.84	-	-	-	32.77	-	-	-	16.35	-	14.25	-
3	2.95	-	-	-	15.09	-	12.84	-	3.62	0.25	-	-	16.32	0.47	2.84	-	-	-	11.25	-
4	-	-	0.78	-	1.72	-	4.46	0.29	2.95	0.44	-	-	5.07	-	3.00	-	7.87	-	-	-
5	0.23	0.08	0.40	0.60	1.80	-	3.12	0.22	0.27	0.24	0.30	-	1.45	-	0.85	0.29	5.07	-	0.58	-
6	0.002	0.24	0.04	0.13	0.10	-	0.42	0.17	0.33	0.08	0.03	0.56	1.74	0.53	0.69	0.10	1.23	-	0.23	-
7	0.06	0.10	0.14	0.21	0.46	0.55	0.07	0.10	0.32	0.05	0.05	0.25	0.05	0.08	0.45	0.10	0.02	0.33	0.05	0.19
8	0.02	0.01	0.06	0.04	0.003	0.18	4E-4	0.17	0.10	0.08	0.26	0.02	0.09	0.06	0.15	0.05	0.34	0.10	0.18	0.16
9	0.02	0.06	0.01	0.02	0.04	0.02	0.03	0.10	0.14	0.01	0.26	0.05	0.15	0.13	0.12	0.04	0.36	0.20	0.16	0.03
10	0.04	0.03	0.09	0.02	0.02	0.02	0.06	0.03	0.04	0.01	0.25	0.01	0.15	0.03	0.18	0.02	0.39	0.19	0.12	0.05

S2.2 Friedman and Nemenyi tests on relative errors and relative standard deviations

Table S6: Results of a Nemenyi post-hoc test performed on the relative errors (REs) and relative standard deviations (RSDs) of standard HSQC, Q-HSQC and QUIPU-HSQC computed over all metabolites. Note that the preceding Friedman test resulted in significant results for both REs and RSDs $p < 0.05$.

	standard HSQC		Q-HSQC	
	RE	RSD	RE	RSD
Q HSQC	0.043	0.370	-	-
QUIPU HSQC	$5 \cdot 10^{-7}$	$2.6 \cdot 10^{-14}$	0.013	$1.0 \cdot 10^{-12}$

Table S7: Results of Friedman and Nemenyi post-hoc tests performed on the relative errors (REs) and relative standard deviations (RSDs) of individual metabolites computed for standard HSQC (St), Q-HSQC (Q) and QUIPU-HSQC (QU).

	Friedman		Nemenyi					
	RE	RSD	RE			RSD		
			St-Q	St-QU	Q-QU	St-Q	St-QU	Q-QU
alanine	0.223	0.069	0.970	0.900	0.970	0.173	0.001	0.173
creatinine	0.018	0.042	0.110	0.110	1.000	0.781	0.002	0.020
glycine	0.180	0.039	0.110	0.370	0.780	0.781	0.020	0.002
taurine	0.135	0.006	0.037	0.644	0.261	1.000	0.020	0.020
betaine	0.008	0.005	0.896	0.065	0.173	0.503	0.001	0.037
acetic acid	0.135	0.247	0.170	0.010	0.500	0.896	0.010	0.037
ethanol-amine	0.018	0.015	0.01	0.500	0.170	1.000	0.110	0.110
TMAO	0.002	0.009	0.109	0.0002	0.109	0.896	0.001	0.005
histidine	0.030	0.050	0.065	0.037	0.973	0.372	0.0002	0.020
citric acid	0.006	0.050	1.000	0.110	0.110	0.644	0.0004	0.010

S2.3 Urine spike-in

Table S8: 21 amino acids were added at two different concentrations to a sample of human urine to analyze the performance of the Q-HSQC in a real world setting. Note that the first line contains the values determined in the blank sample, while the the next two lines contain the values obtained by Q-HSQC after subtraction of blank values. Note some of the blank values were in the low µM range and, therefore, not amenable by 2D NMR. Consequently, all blank values were determined by 1D NMR employing the Chenomx 8.6 software suite (Chenomx Inc., Edmonton, Canada).

mM	Ala	Arg	Asn	Asp	Glu	Gln	Gly	Hipp	His	Ile	Leu	Lys	Met	Orn	Phe	Pro	Ser	Thr	Trp	Tyr	Val
0.0	0.080	0.102	0.022	0.033	0.138	0.187	0.114	1.596	0.013	0.009	0.008	0.015	0.012	0.040	0.585	0.106	0.125	0.078	0.015	0.032	0.014
0.3	0.267	0.229	0.315	0.591	0.287	0.309	0.314	0.340	0.254	0.273	0.333	0.249	0.263	0.222	0.291	-	0.586	0.205	0.384	0.430	0.242
0.6	0.581	0.569	0.616	0.571	0.534	0.552	0.577	0.682	0.773	0.600	0.754	0.549	0.564	0.652	0.686	0.521	0.712	0.437	0.678	0.695	0.581

References

1. Mauve, C.; Khlifi, S.; Gilard, F.; Mouille, G.; Farjon, J. Sensitive, highly resolved, and quantitative 1H-13C NMR data in one go for tracking metabolites in vegetal extracts. *Chemical Communications* **2016**, 52, 6142–6145.
